# Announcement: Fifth Eurasia Conference on Chemical Sciences

**DOI:** 10.1155/MBD.1996.165

**Published:** 1996


### FIFTH EURASIA CONFERENCE ON CHEMICAL SCIENCES

### Guangzhou (Canton), China, December 10-14, 1996

Sponsored by
the Chinese Chemical Society, the National Natural Science Foundation of China and the Government of Guangdong Province

Hosted by
Zhongshan (Sun Yatsen) University and Guangdong Provincial Chemical Society

### GENERAL INFORMATION

### INTRODUCTION

Since its inception at Bangkok, Thailand (1988), the Eurasia Conference on Chemical Sciences has attracted a large number of outstanding scientists together not only from the supercontinent of Eurasia but also from other continents for the enhancement of scientific cooperation and exchanging views on the development of chemical research. The conference has been held biennially at Seoul, South Korea (1990), Bangkok, Thailand (1992), and Kuala Lumpur, Malaysia (1994)

The fifth EuAsC2S-5 will take place at White Swan Hotel in Guangzhou, China from Tuesday 10 December to Saturday 14 December 1996, with forgathering on Monday 9 December and departing on Sunday 15 December.

The Organizing Committee has made every effort to present a program providing creative scientific insights and new discoveries as well as cultural and social activities.

The conference will begin with a welcome reception on Monday evening 9 December, and scientific sessions will begin on Tuesday morning 10 December and continue through Saturday afternoon 14 December except Thursday 12 December, which has been set aside for an organized excursion. The conference banquet has been arranged on Friday 13 December.

### INTERNATIONAL ORGANIZING COMMITTEE

**Chairman:**
Prof. Jan Reedijk *Nefherlands*
**Members:** Prof. Ivano Bertini, Italy; Prof. Mu Shik Jhon, South Korea; Prof. Hitoshi Ohtaki, Japan; Prof. B. Michael Rode, Austria; Prof. A. Geoff Sykes, UK; Prof. John Webb, Australia
**Emeritus Member:** Prof. Salag Dhabanandana, Thailand
**Secretary:** Prof. Youngkyu Do, South Korea

### INTERNATIONAL ADVISORY BOARD

Prof. N. Al-Shatti, Kuwait; Prof. Gilbert Balavoine, France; Prof. Kalman Burger, Hungary; Prof. Jean T. Clerc, Switzerland; Prof. Fabian M. Daurit, Philippines; Prof. Harry B. Gray, USA; Prof. Karl Heinzinger,Germany; Prof. Bertil Holmberg, Sweden; Prof. N. Katsaros, Greece; Prof. Kean H. Khoo, Brunei; Prof. V. Krishnan, India; Prof. Pierre Laszlo, France; Prof. Jean-Marie Lehn, France; Prof. Yizhak Marcus, Israel; Prof. Ulrich Mayer, Austria; Prof. M. Mosihuzzaman, Bangladesh; Prof. Duy Cuong Nguyen, Vietnam; Prof. Barry N. Noller, Australia; Prof. Charmian J. O'Connor, New Zealand; Prof. Andrea Scozzafava, Italy; Prof. Bambang Setiadji, Indonesia; Prof. Helmut Sigel, Switzerland; Prof. M. Mohinder Singh, Malaysia; Prof. Roma Tauler, Spain; Prof. Kui Wang, China; Prof. David R. Williams, UK; Prof. Nicola D. Yordanov, Bulgaria

**NATIONAL ADVISORY BOARD**
Yong-Zheng; Hui Xiang Liang; Shang-An Lin; Zhong-He Lu; Ao-Qing Tang; Xun-Zhang Wang; Liang Wu; Dong-Sheng Yan; Cun-Hao Zhang; Dao-Ben Zhu; Li-Lan Zhu

**NATIONAL ORGANIZING COMMITTEE**
**Chairman**: Prof. Kui Wang, Beijing
**Vice Chairman**: Prof. Yuan-Tong Xu, Guangzhou
**Co-Chairman and.General Secretary:** Prof. Liang-Nian Ji, Guangzhou
**Vice General Secretary:** Dr. Zong-Wan Mao, and Assoc. Prof. Le-Lan Huang, Guangzhou
**Members:** Chun-Li Bai; Jian Chen; Guang-Min Cong; Yi-Yao Ni; Xing-Guo Peng; Chung-Kwong Poon; Xi-Bai Qiu; Lei Wang; Shi-Du Zeng; Pei-Heng Zhong; Guang-Mei Zhu

**SCIENTIFIC PROGRAM COMMITTEE**
Min-Bo Chen; Xiao-Ming Chen; Li-Xin Dai; Bei-Sheng Kang; Zhuo-Mei Li; Yuan-Fang Liu; Jin-Yuan Mo; Jia-Zan Ni; Xiao-Zeng You; Ru-Qin Yu; Yan-Sheng Yang; Hang-Min Zeng; Long-Mei Zeng; Zhan-Xia Zhang; Xin-Sheng Zhao

**LOCAL EXECUTIVE COMMITTEE**
Rui-Hua Dai; Wan-Shu Han; Qi-Rui He; Ji-Sheng Huang; Zi-He Li; Han-Biao Liu; Hui-Ying Yang; Run-Jian Zhang; Zhi-Yao Zhang

### DATE AND VENUE
Date: December 10 (Tue.) - 14 (Sat.) 1996
Venue: White Swan Hotel, Shamian Island, Guangzhou, China, Tel: 0086-20-8886968, Fax: 0086-20-8861188

### OFFICIAL LANGUAGE
English. No translation facilities will be provided.

### INVITATION
The organizing committee will be glad to forward an official invitation letter upon request. It should be noted, however, that this invitation does not imply any financial support. However, please note that participants from developing countries can apply to the United Nations Educational Scientific and Cultural Organization (UNESCO) office in their home countries for travel funds to take part in EuAsC2S-5 and to a topic related to "natural products".

### PASSPORT AND VISA
Every foreign visitor entering China must be in possession of a valid passport. Form D, obtainable from Prof. Liang-Nian Ji, should be sent to: Mr. Xi-Bai Qiu, The Secretary of EuAsC2S-5, Chinese Chemical Society, P. O. Box 2709, Beijing 100080, China, before October 1, 1996 who will send a formal invitation for visa application. For those visitors whose country has no diplomatic relation with the People's Republic of China, the Visa Application form should be sent to Mr. Xi-Bai Qiu no later than September 1, 1996.

### TRANSPORTATION
Direct flights to Guangzhou are restricted from a few cities in Asian countries. Usually a convenient access to Guangzhou is via Hong Kong, there are several runs of through trains and flights to and from between Hong Kong and Guangzhou every day. It takes approximately 20 minutes by air and about 2 hours by train from Hong Kong to Guangzhou. International/domestic flights are also available between Shanghai, Xiamen (Amoy), Beijing and other Chinese port cities and Guangzhou. Persons with the sign "EuAsC2S-5" will meet the participants at the Guangzhou Baiyun Airport or at the Guangzhou Railway Station during the following time: 8:00-23:00 December 9th. Free limousine buses run intermittently will be provided by the Organizer to transport participants to the White Swan Hotel.

Participants arriving at other times should make their way to the White Swan Hotel by taxi, it is convenient and inexpensive. The organizer will make arrangements to meet you at the registration desk at any time during the night (Dec. 9-10).

### SCIENTIFIC PROGRAM

### OPENING CEREMONY

The Opening Ceremony will be held 8:50-9:15 on Tuesday morning 10 December 1996. The official opening will be declared by Vice-Governor of Guangdong Province or the president of Zhongshan University while the ceremony will be chaired by Prof. Liang-Nian Ji, the Co-Chairman of the Committee, and welcome addresses will be delivered by Prof. Jan Reedijk, the Chairman of International Organizing Committee and by Kui Wang, the Chairman of National Organizing Committee.

The program will be organized to consist of seven themes:

1. Energy sources and chemistry
2. Environment protection
3. Chemical problems in life sciences
4. Natural products
5. Chemistry of Materials
6. Theoretical and Computational Chemistry
7. Analytical Chemistry and its Methodology

Four plenary lectures will be organized as the leading talks which will be expanded by in the following 12 platform sessions and minisymposia respectively. Twelve special topics will be discussed in minisymposia. For each minisymposium, one key lecture plus 7 oral presentations will be invited. The 12 topics of sessions, minisymposia and poster presentations include:

MS1 Bioremediation
MS2 Environmental and Analytical Chemistry
MS3 Chemical Solution to Problems in Life Sciences
MS4 Organic Synthesis and Chiral Catalysis
MS5 Natural Products and Biopolymers
MS6 Nano-materials with Special Attention to Coordination Materials
MS7 Theoretical, Computational and Modeling Chemistry
MS8 Solution Coordination Chemistry and Transfer of Chemicals Between Solvents
MS9 Molecular Structure under Extreme Conditions in Solution
MS10 Traditional Drugs
MS11 Chemometrics
MS12 Bioinorganic Catalysis

Four plenary lectures will be presented as the leading talks every morning of the four days of Scientific Sessions. The following plenary speakers have confirmed their participation:

**Jean-Marie Lehn**, Univ. Louis Pasteur 4, France: Supramolecular chemistry
**K. Wieghardt,** Max-Planck-lnst. für Strahlenchemie, Germany: Modeling the binuclear active sites in some metallo-proteins chemistry: successes, challenges and failures
**H. Sakurai**, Tokyo University of Science, Japan: Group 14 organometallic chemistry
**Ru-Ren Xu**, Jilin Univ, Jilin: Progress in microporous materials

There will be 12 concurrent sessions and minisymposia following the plenary talks; 3 sessions and 3 minisymposia in parallel are presented every day with 2 keynote lectures being presented in each session and a key lecture in each minisymposium. The scientists who have accepted to speak and the title of their lectures are as follows:

**N. C. Yang**, The Univ. of Chicago, USA: Supramolecular chemistry of helical peptide bundles
**E. Kimura**, Hiroshima Univ., Japan: Development of new macrocyclic polyamine complexes for controlling gene expression
**T. Ito**, Tohoku Univ, Japan: Pyrazine-bridged oligomers of trinuclear Ru complexes with $Ru_3$ core: reversible multi-step/multi-electron redox behavior
**T. Yamaguchi**, Fukuoka Univ., Japan: Recent development in structural approach to solution chemistry

**U. Mayer**, Technical Univ. of Vienna, Austria: Chemical modified surfaces: some recent developments
**M. S. Jhon**, Korea Advanced Inst. of Sci. and Tech., Korea: Structure of water and proteins
**K. Burger**, A. Jozself Univ., Hungary: Coordination and H-bond formation equilibria in submicroscopic (nanosize) droplets: Mössbauer and NMR studies
**C. M. Che**, Univ of Hong Kong, H.K.: Organic oxidation by Ru-oxo complexes, catalytic and asymmetric
**J. Slanina**, Energieonderzoek Centrum Nederland, Netherlands: Uncertainties in cycles of greenhouse gases and in the radioactive balance of the earth
**H. Sigel**, Univ of Basel, Switzerland: Metal-lon interactions of antiviral nucleotide analogues in solution
**J. K. Barton**, California Inst. of Tech., USA: DNA damage and repair by transition metal complexes
**P. Dervan**, Califomia Inst. of Tech., USA: Molecular design for recognition of DNA
**A. G. Sykes**, The Univ. of Newcastle Upon Tyne, UK: Free radicals in protein reactions
**O. Yamauchi,** Nagoya Univ., Japan: Stacking interactions in metal complexes of biological molecules
**N . Hadjiliadis**, Univ of loannina, Greece: The interaction of heavy metal with small peptides containing cysteine and/or histidine
**Y. Fukuda**, Ochanomizu Univ, Japan: Chromotropic behaviors of transition-metal complexes
**Xiao-Yuan Li,** H.K. Univ. of Sci. and Tech., H. K.: Biological Problems of nitric oxide: a comprehensive chemical approach
**A. Imamura**, Hiroshima Univ., Japan: Local modeling method for energy lines in linear polymers
**E. Pungor**, Tech. Univ of Budapest, Hungary: lons selective electrodes and their mechanism and application
**T. Ogawa**, Kyushu Univ., Japan: Surface of water as studied by laser two-photon ionization and second harmonic generation
**S. Iwata**, Inst. of Molecular Science, Japan: Structures, spectroscopies and reaction dynamics of molecular clusters
**Dae Dong Sung**, Dong-A Univ., Korea: Carborane chemistry
**H. Hosaya**, Ochanomizu Univ., Japan: The recent advances in mathematical chemistry especially in the application of graph theory to chemistry
**J.-J. Godfroid**, Univ. Paris 7, France: Platelet-activating factor: from natural products to molecular modelization
**W. D. Horrocks, Jr**., The Pennsylvania State Univ., USA: Supramolecular coordination chemistry in aqueous solution
**D. Chapman**, The Royal Free Hospital, UK: Biomembranes, ion-channel and novel biomaterials
**E. S. Yeung**, lowa State Univ., USA: Single-molecule detection and characterization by laser spectroscopy
**J. P. Doucet**, Univ. Paris 7, France: Neural networks as new tools in molecular modeling
**Xin-Sheng Zhao**, Beijing Univ., Beijing: The syplectic quantum propagation
**Zhong-Xian Huang**, Fudan Univ., Shanghai: Study on site-directed mutagenesis and structure, functions of electron transfer protein—cytochrome b5
**Qi Xue**, Nanjing Univ., Nanjing: Complexation of thiophene in solvated Lewis acids and the formation of a highly anisotropic conducting polymer
**Li-Xin Dai**, Shanghai Inst. of Org. Chem., Shanghai: Stereoselective synthesis of cyclopropanes, oxiranes and aziridines
**Chun-Li Bai,** Beijing Inst. of Chem., Beijing: Characterization of the new structure of DNA in solution
**Xi-Yan Lu**, Shanghai Inst. of Org. Chem., Shanghai: Some new aspects on Pd(ll) catalyzed organic reactions
**Liang Huang**, Inst. of Pharmacology, Beijing: Chemical and biological studies of clausenamides isolated from traditional Chinese medicine, leaves of clausena lansium
**Xiao-Zeng You**, Nanjing Univ., Nanjing: Study on molecular-based non-linear optical materials

### MINISYMPOSIA

Minisymposia have been arranged every morning and afternoon of the conference program. The following organizers of the minisymposia have been confirmed:

MS 1: Convenor **J. Webb**, Murdoch Univ., Australia; Co-convenor **A. Scozzafava**, Univ. of Florence, Italy; Local convenor **Zhan-Xia Zhang**, Zhongshan Univ., Guangzhou
MS 2: Convenor **E. Eyring**, Univ. of Utah, USA; Local convenor **Ben-Li Huang**, Xiamen Univ., Xiamen
MS 3: Convenor **D. R. Williams**, Univ. of Wales, UK; Local convenor **Yu-Fen Zhao**, Tsinghua Univ, Beijing

MS 4: Convenor **G. Balavoine**, CNRS, France; Local convenor **Long-Mei Zeng**, Zhongshan Univ., Guangzhou
MS 5: Convenor **R. M. Letcher,** Univ. of Hong Kong; Local convenor **Li-He Zhang**, Beijing, Medical Univ., Beijing
MS 6: Convenor **Y. Sasaki,** Hokkaido Univ., Japan; Local convenor **Ming Jiang**, Fudan Univ., Shanghai
MS 7: Convenor **B. M. Rode**, Univ. Insbruck, Austria; Co-convenor **Min-Bo Chen**, Inst. of Org. Chem., Shanghai; Local convenor **Yuan-Sheng Jiang**, Nanjing Univ., Nanjing
MS 8: Convenor **Y. Marcus**, The Hebrew of Univ. Jerusalem, Israel; Co-convenor **B. Holmberg**, Lund Univ., Sweden; Local convenor **Xin-Quan Xin**, Nanjing Univ., Nanjing
MS 9: Convenor **T. Radnai**, Central Res. Inst. for Chem., Hungary ; Co-convenor **H. Ohtaki**, Ritsumeikan University, Japan ; Local convenor **Zheng Xu**, Nanjing Univ., Nanjing
MS 10: Co-convenor **S. Kokpol**, Chulalongkom Univ., Thailand; Local convenor **Jing-Yu Su**, Zhongshan Univ., Guangzhou
MS 11: Convenor **R. Tau!er,** Univ. of Barcelona, Spain; Local convenor **Ru-Qing Yu**, Hunan Univ., Hunan
MS 12: Convenor **I. Bertini,** Univ. of Florence, Italy; Co-convenor **S. I. Chan**, Califomia Inst. of Tech., USA; Local convenor **Jia-Zan Ni**, Inst. of Applied Chem., Changchun

### POSTERS

Poster Sessions have been arranged for 10:00-1 1:00 every morning and 14:00-15:30 every afternoon. Each poster must be within size of 1,2 m high x 1,0 m wide space

### CALL FOR CONTRIBUTED PAPERS

Contributed papers are accepted in the form of either oral or poster presentations, which will carry equivalent scientific status in the program and in the resulting publication. From the abstracts submitted in due time, several works will be invited to be presented orally in a certain minisymposium. Each minisymposium is limited to a 10 minutes convenor's introduction, a key lecture of 35+5 minutes five (25+5) minutes invited lectures and two 20 minutes contributed papers. Each plenary lecture and keynote lecture will last 50 and 25+5 minutes respectively. For each speaker, the 35 mm slide projector and overhead projector will be available. The slides should be mounted on a 50mmx50mm frame. Abstracts will only be accepted upon receipt of registration fee from the first author or correspondence author. Each participant may submit only one abstract as first (i. e. presenting) author, but may be a co-author of abstracts with other presenters. The best poster presented will receive an award.

### OTHER INFORMATION

### SATELLITE MEETINGS

### Biomimetic and Asymmetric Oxidation Workshop

Hong Kong, Dec. 16-17, 1996 (a two-day workshop). For further information contact:
Prof. Chi-Ming Che, Department of Chemistry, University of Hong Kong, Pokfulam Road, Hong Kong, Tel: 852-2859-7919, Fax: 852-2857-1586, E-mail: cmche@hkucc.hku.hk

### Molecular Modeling on both Life Science and Material Science Workshop

Guangzhou, Dec. 16-19, 1996 (a four-day workshop)
Dec.16: Seminar Day

- Overview the development and applications of Molecular Modeling
- Application stories in Life Science
- Application stories in Material Science
- Some outstanding scientists will present their work.

Dec. 17: Introduction to Molecular Modeling

- Demo and Hands on.

Dec. 18: How do you apply molecular modeling in life science
• Drug Design
• Combinatorial Chemistry
• Homology
• Reaction Mechanism
• Interaction between receptor and inhibitor etc.
Dec. 19: How do you apply molecular modeling in material science
• Polymer, Solid State, Electric, Optical, magnetic properties of materials
• Computer aided material design, etc.

The workshop will be supported by Molecule Simulation Inc. Its registration fee will be quite low for all participants. If the workshop aroused the interest of you, please contact Prof. Liang-Nian Ji.

**MISCELLANEOUS INFORMATION**

Zhongshan University is situated by the Pearl River in Guangzhou, which is one of the most important centers of international commerce in south China and also has a history of more than 2,800 years. In December temperature in Guangzhou is in range of 10 °C (50°F) to 20°C (68°F).

### CORRESPONDENCE

All correspondence should be addressed to:

Prof. Liang-Nian Ji
General Secretary, EuAsC2S-5 1996
Biotechnology Research Center
Zhongshan (Sun Yatsen) University
Guangzhou 510275, P. R. China
Tel: 86-20-4185461 Fax: 86-20-4184737 (Before June 8, 1996)
Tel: 86-20-84185461 Fax: 86-20-84184737 (After June 8, 1996)
E-mail: cesjln@zsulink.zsu edu.cn

**IMPORTANT TIME DEADLINE OF RETURN**

| | |
|---|---|
| 1. Abstract submission | Before June 15, 1996 |
| 2. Registration Form and Early Registration Fee | BeforeAugust 1, 1996 |
| 3. Hotel Registration Form | Before August 1, 1996 |
| 4. Room Deposit Fees | Before November 1, 1996 |
| 5. Biographical Data Sheet | Before August 1, 1996 |

